# Attenuation of Antibody Titers from 3 to 6 Months after the Second Dose of the BNT162b2 Vaccine Depends on Sex, with Age and Smoking Risk Factors for Lower Antibody Titers at 6 Months

**DOI:** 10.3390/vaccines9121500

**Published:** 2021-12-18

**Authors:** Yushi Nomura, Michiru Sawahata, Yosikazu Nakamura, Ryousuke Koike, Otohiro Katsube, Koichi Hagiwara, Seiji Niho, Norihiro Masuda, Takaaki Tanaka, Kumiya Sugiyama

**Affiliations:** 1 Department of Respiratory Medicine and Clinical Immunology, National Hospital Organization Utsunomiya National Hospital, Utsunomiya 329-1193, Japan; uc.nomura@gmail.com (Y.N.); ryou.k.1207@gmail.com (R.K.); 5okatsube@gmail.com (O.K.); sugiyama@dokkyomed.ac.jp (K.S.); 2 Department of Pulmonary Medicine and Clinical Immunology, Dokkyo Medical University, Shimotsuga 321-0293, Japan; siniho@dokkyomed.ac.jp; 3 Department of Medicine, Division of Pulmonary Medicine, Jichi Medical University, Shimotsuke 329-0498, Japan; hagiwark@me.com; 4 Department of Public Health, Jichi Medical University, Shimotsuke 329-0498, Japan; nakamuyk@jichi.ac.jp; 5 Department of Surgery, National Hospital Organization Utsunomiya National Hospital, Utsunomiya 329-1193, Japan; norim0507@gmail.com; 6 Department of Orthopaedic Surgery, National Hospital Organization Utsunomiya National Hospital, Utsunomiya 329-1193, Japan; tanaka.takaaki.vh@mail.hosp.go.jp; 7 Saitama Medical Center, Department of Respiratory Medicine and Clinical Immunology, Dokkyo Medical University, Shimotsuga 321-0293, Japan

**Keywords:** SARS-CoV-2, viral infection, clinical epidemiology

## Abstract

Objective: We aimed to determine antibody titers at six months and their percentage change from three to six months after the second dose of the BNT162b2 coronavirus disease 2019 (COVID-19) mRNA vaccine (Pfizer/BioNTech) and to explore clinical variables associated with titers in Japan. Methods: We enrolled 365 healthcare workers (250 women, 115 men) whose three-month antibody titers were analyzed in our previous study and whose blood samples were collected 183 ± 15 days after the second dose. Participant characteristics, collected previously, were used. The relationships of these factors with antibody titers at six months and percentage changes in antibody titers from three to six months were analyzed. Results: Median age was 44 years. Median antibody titer at six months was 539 U/mL. Older participants had significantly lower antibody titers (20s, 752 U/mL; 60s–70s, 365 U/mL). In age-adjusted analysis, smoking was the only factor associated with lower antibody titers. Median percentage change in antibody titers from three to six months was −29.4%. The only factor significantly associated with the percentage change in Ab titers was not age or smoking, but sex (women, −31.6%; men, −25.1%). Conclusion: The most important factors associated with lower antibody titers at six months were age and smoking, as at three months, probably reflecting their effect on peak antibody titers. However, the only factor significantly associated with the attenuation in Ab titers from three to six months was sex, which reduced the sex difference seen during the first three months. Antibody titers may be affected by different factors at different time points.

## 1. Introduction

The BNT162b2 vaccine (Pfizer/BioNTech), which was the first coronavirus disease 2019 (COVID-19) mRNA vaccine to be administered to healthcare professionals in Japan, starting in February 2021, is expected to help to prevent both the onset [1] and progression of COVID-19. To obtain the maximum efficacy of vaccination with reduced adverse effects and to optimize and individualize the vaccination regimen, we need to record the exact chronological changes in real-world antibody (Ab) titers following BNT162b2 vaccination and to investigate the relationships of recipients’ medical histories and demographic characteristics with Ab titers.

Factors associated with the percentage change in Ab titers in the first few months after the second of the two BNT162b2 doses could differ from those associated with the peak Ab titers immediately after the second of the two BNT162b2 doses. The median titer shortly after the full schedule of this vaccine in Japan has been reported to be 2060 U/mL (interquartile range [IQR], 1250–2650 U/mL) [2], which is similar to the median titer reported in Italy (1975 U/mL; IQR, 895–3455 U/mL) [3]. Various factors, including older age [2,4,5,6], male sex [2], ethnicity [7], social condition [7], obesity [8,9], smoking habit [6,9], drinking habit [2], hypertension [9], cancer [10,11], and use of immunosuppressive drugs [2], have been reported to reduce the Ab titers obtained shortly after the second dose of the BNT162b2 vaccine. In Japan, older age, male sex, and drinking habit are reported risk factors for a lower peak value of the Ab titer [2].

In our previous cohort study of the serological Ab titers of 378 healthcare workers at 3 months after the second dose of the BNT162b2 vaccine [12] in Tochigi prefecture, Japan, these titers ranged from 3 to 5790 U/mL, and the median (IQR) Ab titer was 764 (423–1140) U/mL, which was much lower than the above-mentioned value obtained shortly after the second inoculation. We thus determined that age and smoking status are predictors of Ab titers three months after the second dose of the BNT162b2 vaccine in Japan. However, the medium-term attenuation percentages of Ab titers several months after the second BNT162b2 dose and the relationship of these percentages with clinical background and demographic characteristics are still not known.

Against this background, we analyzed Ab titers at six months and their percentage change from three to six months after the second dose of the BNT162b2 vaccine and explored the clinical variables associated with these parameters in Japan. To investigate the Ab titers at three and six months, it is important to predict the period of effective Ab titers.

## 2. Methods

### 2.1. Population and Study Design

In this single-center prospective observational study, we recruited healthcare workers whose blood samples were collected 183 ± 15 days after the second of two BNT162b2 vaccine inoculations (Pfizer/BioNTech) administered 3 weeks apart in February and March 2021 in the National Hospital Organization Utsunomiya National Hospital in Tochigi prefecture, Japan.

Initially, we recruited 378 participants whose Ab titers 3 months after the second dose of the BNT162b2 COVID-19 vaccine were already analyzed in our previous study [12]. Their medical histories and demographic characteristics were recorded in that study using a structured self-report questionnaire [12]. Vaccination was voluntary, and all vaccinated individuals were included as participants. However, we excluded 13 participants, namely, 5 who had already moved from our hospital, 2 who were absent, 3 who declined to participate, and 3 whose blood sampling confirmed the presence of Abs against the nucleocapsid proteins for severe acute respiratory syndrome coronavirus 2 (SARS-CoV-2). Finally, we enrolled 365 healthcare workers (250 women, 115 men). After informed consent was obtained, we administered a questionnaire that covered personal information, such as smoking, drinking, complications, and medication.

Blood samples collected 183 ± 15 days after the second inoculation were used to measure total Ab titers against the SARS-CoV-2 spike antigen using a commercially available electrochemiluminescence immunoassay (ECLIA) (Elecsys® Anti-SARS-CoV-2 RUO; Roche Diagnostics) [13]. The relationships between Ab titers against the SARS-CoV-2 spike antigen and clinical and lifestyle characteristics were analyzed. In age-adjusted analysis, individual Ab titers were recalculated by subtracting the median Ab titer of the corresponding age group from an individual’s Ab titer. For example, an age-adjusted Ab titer for an individual in his/her 20s was calculated as follows: individual Ab titer—median Ab titer for participants in their 20s.

In addition, we calculated the percentage change in Ab titers from 3 to 6 months after the second dose of the BNT162b2 COVID-19 vaccine. Individual percentage changes in Ab titers were calculated as follows: percentage change = ((Ab titer 6 months after the 2nd dose − Ab titer 3 months after the 2nd dose [12])/Ab titer 3 months after the 2nd dose) × 100 (%). Then, the relationships between these percentage changes in Ab titers against the SARS-CoV-2 spike antigen and clinical and lifestyle characteristics were analyzed.

The Ethics Committee of National Hospital Organization Utsunomiya National Hospital (No. 03-01; 19 April 2021) approved this study, and written informed consent was obtained from all participants before enrollment.

### 2.2. Data Analysis

Nonparametric continuous data are expressed as the median with the IQR. Categorical data are presented as absolute numbers and relative frequencies (*n*, %). To calculate Spearman’s rank correlation coefficient and perform the Mann–Whitney *U* test, we used Statistical Package for the Social Sciences (SPSS version 25). We considered *p* values less than 0.05 to be statistically significant.

## 3. Results

### 3.1. Study Population

In total, 365 healthcare workers (250 women, 115 men) were enrolled in this study. Their baseline characteristics are summarized in Table 1. Briefly, the median (IQR) age and mean (SD) age of the participants was 44 (32–54) years and 43.9 (±13.5) years, respectively. Nurses (*n* = 171) and physicians (*n* = 34) comprised 56.2% of the study population.

### 3.2. Distribution of Ab Titers against the SARS-CoV-2 Spike Sntigen 6 Sonths after the Second Dose of the BNT162b2 COVID-19 Vaccine by Age and Sex

**Figure 1:**
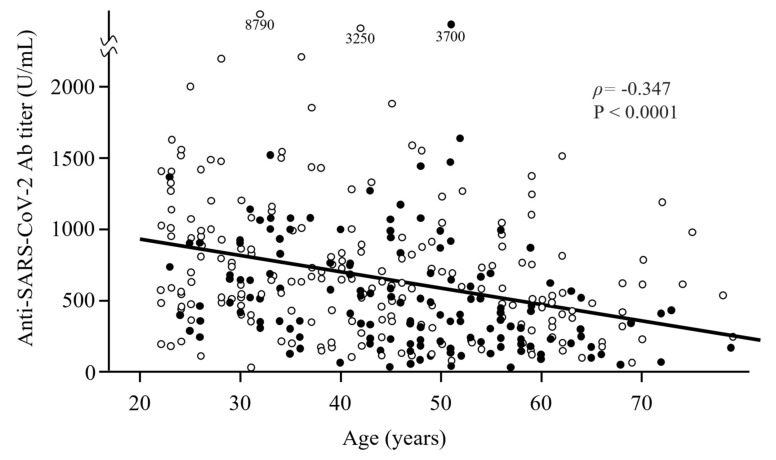
Scatter plot of the distribution of Ab titers 6 months after the second vaccine dose according to age and smoking status. Older participants had significantly lower Ab titers. Closed and open circles show ever-smokers and never-smokers, respectively. Ever-smokers had lower Ab titers than never-smokers.

The median (IQR) Ab titer against the SARS-CoV-2 spike antigen 6 months after vaccination with the BNT162b2 COVID-19 vaccine was 539 (309–872) U/mL. Older participants had significantly lower SARS-CoV-2 Ab titers (correlation coefficient ρ = −0.347) (Table 1 and Figure 1). Ab titers tended to decrease in both men and women as participants’ age increased from their 20s to 70s. Median antibody titers of men in their 20s, 30s, 40s, 50s, and 60s–70s were 823, 648, 485, 335, and 432 U/mL, respectively, whereas median Ab titers of women in their 20s, 30s, 40s, 50s, and 60s–70s were 748, 641, 560, 490, and 318 U/mL, respectively.

### 3.3. Relationship between the Ab Titers against the SARS-CoV-2 Spike Antigen 6 Months after Vaccination and Risk Factors

We first performed univariate analyses to identify factors associated with serum Ab titers against the SARS-CoV-2 spike protein. The factors significantly associated with lower Ab titers were older age, smoking, and hypertension (Table 1). Ever-smokers tended to have lower Ab titers than never-smokers (Figure 1).

We also analyzed the risk factors for lower Ab titers after adjustment for age because the prevalence of certain factors may differ according to age, such as hypertension. In the age-adjusted analysis, individual Ab titers were recalculated by subtracting the median Ab titer of the corresponding age group from an individual’s Ab titer. Median Ab titers of participants in their 20s, 30s, 40s, 50s, and 60s–70s were 752, 642, 550, 418, and 365 U/mL, respectively. Thus, the age-adjusted Ab titer for an individual in his/her 20s was calculated as “individual Ab titer—752”. After age adjustment, the only factor significantly associated with lower Ab titers was smoking (Table 2). In terms of smoking, the age-adjusted median (IQR) Ab titers were −97 (−277 to 184) and 56 (−182 to 342) in ever-smokers and never-smokers, respectively.

### 3.4. Distribution of the Percentage Change in Ab Titers from 3 to 6 Months after the Second Dose of the BNT162b2 COVID-19 Vaccine by Age and Sex

**Figure 2:**
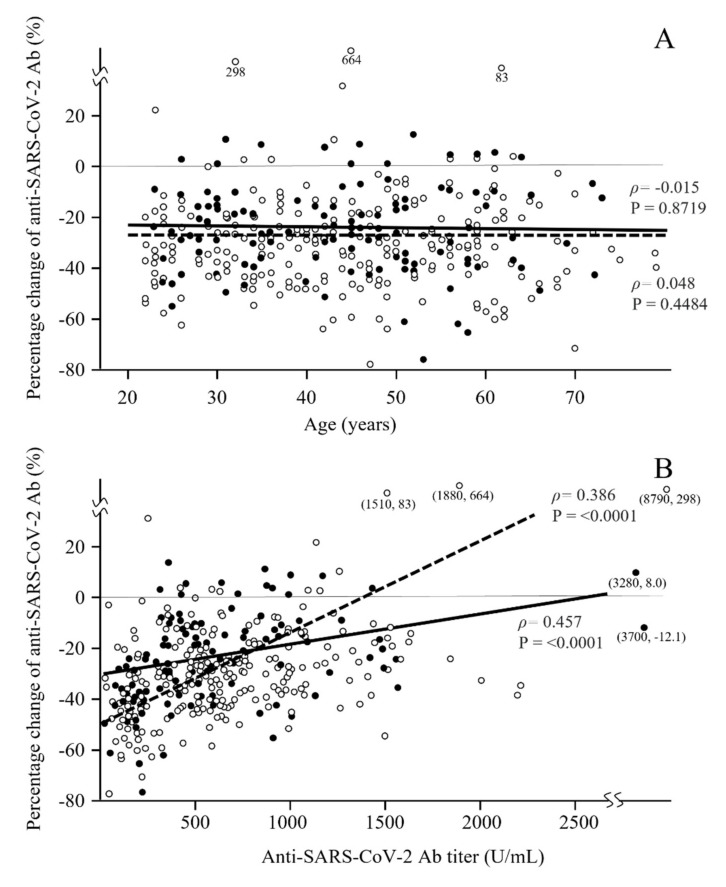
Scatter plot of the distribution of the percentage change in Ab titers from 3 to 6 months after the second dose of the vaccine according to sex. The relationship between the percentage change after the vaccination and age is shown in (**A**), and the relationship between the percentage change and the Ab titer 6 months after the vaccination is shown in (**B**). No significant correlation was observed in (**A**), and age did not affect the attenuation of the Ab titers from 3 to 6 months after the vaccination. However, a significant correlation was observed in (**B**). Closed and open circles and continuous and broken lines show men and women, respectively.

The median (IQR) percentage change in Ab titers against the SARS-CoV-2 spike antigen from three to six months after vaccination was −29.4% (−40.4% to −17.7%). No significant correlation was observed between the percentage change in the anti-SARS-CoV-2 Ab titer and age (correlation coefficient ρ = −0.028) (Table 3 and Figure 2A). Median percentage changes in Ab titers of men in their 20s, 30s, 40s, 50s, and 60s–70s were −24.3, −25.3, −24.0, −33.7, and −14.8 U/mL, respectively, whereas those of women in their 20s, 30s, 40s, 50s, and 60s–70s were −33.8, −31.5, −31.5, −30.0, and −32.6 U/mL, respectively. Surprisingly, the only factor significantly associated with the percentage change in the Ab titer was not age or smoking, but sex (Table 3). In terms of sex, median (IQR) percentage changes in Ab titers were −31.6% (−42.0% to −20.2%) and −25.1% (−36.8% to −12.0%) in women and men, respectively (Table 3), suggesting that Ab titers decrease faster in women than in men.

An additional analysis was performed (Table 4) because smoking was an important risk factor at both three and six months after vaccination and the smoking percentages differed between men and women. For age-adjusted median Ab titers, no significant sex differences were observed in the ever-smoker and never-smoker groups. However, both the male and female groups showed significant differences by smoking status in age-adjusted median Ab titers. On the other hand, no significant differences in the median percentage change in Ab titers by smoking status were observed in the male and female groups. However, both the ever-smoker and never-smoker groups showed significant sex differences in the median percentage change in Ab titers.

The relationship between the percentage changes in Ab titers and the Ab titer 6 months after vaccination was analyzed (Figure 2B). A significant positive correlation was observed, indicating that participants with a lower Ab titer had greater attenuation of the Ab titer.

## 4. Discussion

To our knowledge, this is the first study to report real-world Ab titers against the SARS-CoV-2 spike antigen at six months and the percentage changes in Ab titers from three to six months after vaccination and to identify the factors associated with these parameters from a comprehensive range of clinical and lifestyle characteristics in Japan. Few studies have examined this issue [14,15]. Four important findings were obtained. First, in a cohort with a median (IQR) age of 44 (32–54) years, the median Ab titer six months after the second dose was 539 U/mL. Older participants had significantly lower Ab titers, with an Ab titer about half that of those in their 20s. Second, in an age-adjusted analysis, the only risk factor for a lower Ab titer was smoking. Third, the median percentage change in the Ab titer from three to six months after vaccination was −29.4%. Surprisingly, the only factor significantly associated with attenuation of the Ab titers was not age or smoking, but sex. The median percentage changes in Ab titers were −31.6% and −25.1% in women and men, respectively. Fourth, both male and female participants with a lower Ab titer showed greater attenuation of the Ab titer. This means that the percentage change in Ab titers may be affected by individual differences.

In terms of the first and second findings, the most important factors associated with a low Ab titer six months after the second dose were still age and smoking habit, as seen three months after the second dose, probably reflecting their effect on peak Ab titers. Interestingly, a reversal of the sex difference in Ab titers seen three months after the second dose was observed at six months. In our previous study, the median Ab titer was 764 U/mL three months after the second dose [12]. The median Ab titers were 942 and 1095 U/mL in men and women in their 20s, respectively, but 490 and 519 U/mL in men and women in their 60s–70s, respectively. In the age-adjusted analysis, the only risk factors for a lower Ab titer three months after the second dose [12] were male sex and smoking. However, we concluded that the sex difference may have arisen from the sex difference in the smoking percentage, rather than from biological sex differences. We found several studies reporting an association between smoking and lower Ab titers against both influenza virus [16] and hepatitis B virus [17] after vaccination, although the actual mechanisms are poorly understood.

Regarding the third finding, the only factor significantly associated with the attenuation in Ab titers from three to six months after the second dose was not age or smoking, but sex, which seemingly resulted in a reduction of the sex difference in Ab titers seen three months after the second dose. This suggests that Ab titers decrease faster in women than in men.

Concerning the fourth finding, we hypothesized that participants with a higher Ab titer would show greater attenuation of Ab titers because the percentage change from three to six months and the Ab titer three months after vaccination were significantly higher in women than in men. However, a significant positive correlation was observed between the percentage change in Ab titers and the Ab titer six months after vaccination. The Ab titer at six months after vaccination was associated with the percentage attenuation from three to six months, and participants with a lower Ab titer showed greater attenuation of the titer. We could not find other risk factors, such as age and comorbidities, beyond the above. Although we concluded that the attenuation was affected by individual differences, we will continue to define the risk factors through additional study.

The factors identified to affect Ab titers vary by the time elapsed since vaccination. For peak Ab titers 2–5 weeks after vaccination, the reported risk factors were sex, age, and alcohol intake. Three months after vaccination, age and smoking were risk factors in our study, and the effects of sex and alcohol intake were not observed. Six months after vaccination, age and smoking were still risk factors in our study. However, these factors did not affect the attenuation from three to six months. This means that these factors will strongly affect the Ab titer within three months after vaccination and that the effects will persist six months after vaccination. From three to six months after vaccination, sex and lower Ab titer were risk factors. According to the above results, smoking is the most important factor that could be avoided to maintain a higher Ab titer. Thus, it appears that different factors affect the Ab titer at different time points.

Some limitations and possible sources of bias in this study include the following. First, the participants were limited in number and were all healthcare workers vaccinated at a single national hospital in Tochigi prefecture. Therefore, the results obtained in this study might not be generalizable on a wide scale, or even within Japan. Second, we excluded participants with Abs against nucleocapsid proteins on the presumption that they had previously been infected with COVID-19. However, some of these patients had slight increases in Ab titers against the spike protein but became negative for Ab titers against nucleocapsid proteins. One possibility is that they had not been infected with COVID-19 and that the Ab titers against the spike protein had been increased by individual differences. The other possibility is that the Ab titers against nucleocapsid proteins might have not increased due to a small viral load of SARS-CoV-2, even though they may still have been infected with COVID-19. To determine the chance of exposure to a small amount of SARS-CoV-2 virus, we analyzed Ab titers in participants who worked in the COVID-19 ward (data not shown). However, their Ab titers were not increased. If the excluded participants were by chance exposed to a small amount of SARS-CoV-2 virus, it may have been through daily life and not the COVID-19 ward. In addition, five participants had Ab titers exceeding 3000 U/mL and/or a greater than 80% increase in Ab titers against the spike protein, despite negative Abs against nucleocapsid proteins. They may have been infected with COVID-19, considering that a participant with an Ab titer against spike proteins exceeding 5000 U/mL and without Ab titers against nucleocapsid proteins three months after vaccination had positive Ab titers against nucleocapsid proteins at six months. The participant may have been infected with COVID-19 three months after vaccination and the Abs against nucleocapsid proteins may have increased at a late stage due to the infection. However, we do not have evidence that the above five participants were infected with COVID-19 and we did not exclude them from our analysis.

In conclusion, the most important factors associated with a low Ab titer six months after the second dose were age and smoking habit, as seen three months after the second dose, probably reflecting the effects of these factors on peak Ab titers. However, the attenuation of Ab titers from three to six months after the second BNT162b2 dose depended not on age or smoking, but on sex. A significant attenuation of Ab titers from three to six months was solely observed in women and resulted in a reduction of the sex difference in the Ab titer seen at three months. Further studies of the associations between Ab titers and the comprehensive medical histories of individuals are needed to establish a more personalized approach to vaccination involving earlier boosters, different schedules, or different types of vaccines.

## Figures and Tables

**Table 1:** Participants’ baseline characteristics (*N* = 365).

Variable	Total	Ab Titer at 6 Months, Median (IQR), U/mL	Correlation Coefficient *ρ*	*p*-Value
Age, median (IQR), *y*	44 (32–54)		−0.347	<0.0001 ^#^
Sex (M/F), *n*	115/250	482 (305 to 865)/572 (316 to 884)		0.1970 *
Body mass index, median (IQR), kg/m^2^	22.4 (20.2–24.8)		−0.017	0.7446 ^#^
Smoking (ever/never), *n*	149/216	406 (213 to 688)/614 (405 to 957)		<0.0001 *
Current smoker/ex-smoker/unknown	90/45/14	354 (174 to 564)/436 (225 to 894) ^§§^		0.0609 *
		ex-smoker vs. never-smoker		0.0014 *
		current smoker vs. never-smoker		<0.0001 *
Brinkman Index ^§§^, ^§§§^, *n*	129		−0.197	0.0258 ^#^
Drinking, *n*	228/134/3 ^§^	544 (299 to 881)/536 (348 to 868) ^§§^		0.7697 *
Allergy, *n*				
Food	38/292/35 ^§^	515 (307 to 925)/544 (318 to 807) ^§§^		0.7697 *
Drug	37/293/35 ^§^	517 (287 to 759)/549 (321 to 833) ^§§^		0.5858 *
Allergic disease, *n*				
Allergic rhinitis including pollinosis	167/175/23 ^§^	546 (258 to 895)/523 (320 to 792) ^§§^		0.8242 *
Bronchial asthma	43/299/23 ^§^	582 (360 to 885)/522 304 to 837) ^§§^		0.5643 *
Skin allergy including atopic dermatitis	47/295/23 ^§^	641(380 to 1005)/522(303 to 815) ^§§^		0.0710 *
Diabetes mellitus, *n*	12/340/13 ^§^	331 (149 to 589)/540 (318 to878) ^§§^		0.0512 *
Hypertension, *n*	27/325/13 ^§^	356 (195 to 566)/553 (321to 872) ^§§^		0.0327 *
Dyslipidemia, *n*	18/334/13 ^§^	351 (205to 698)/544 (317 to 877) ^§§^		0.0834 *
Collagen disease, *n*	13/332/20 ^§^	303 (126 to 840)/544 (324 to 843) ^§§^		0.2695 *

IQR = interquartile range. *: Mann–Whitney *U* test. ^#^: Spearman’s rank correlation coefficient test. ^§^: yes/no/unknown are shown. ^§§^: unknown is excluded. ^§§§^: ever-smoker only.

**Table 2:** Age-adjusted data of median Ab titers (*N* = 365).

Variable	Ab Titer at 6 Months, Median (IQR), U/mL	Correlation Coefficient *ρ*	*p*-Value
Male/female	−15 (−246 to 241)/8 (−209 to 290)		0.2851 *
Body mass index, median (IQR), kg/m^2^		0.023	0.6647 ^#^
Smoking (ever/never)	−97 (−277 to 184)/56 (−182 to 342)		<0.0001 *
Current smoker/ex-smoker	−205 (−320 to 7)/−72 (−264 to 256)		0.0255 *
	ex-smoker vs. never-smoker		0.0203 *
	current smoker vs. never-smoker		<0.0001 *
Brinkman Index ^§§^, ^§§§^		0.012	0.8935 ^#^
Drinking	3 (−242 to 296)/5 (−205 to 249) ^§^, ^§§^		0.6475 *
Allergy			
Food	18 (−250 to 235)/−2 (−223 to 256) ^§^, ^§§^		0.9604 *
Drug	−4 (−131 to 271)/−1 (−238 to 251) ^§^, ^§§^		0.5858 *
Allergic disease			
Allergic rhinitis including pollinosis	−7 (−240 to 271)/−1 (−223 to 253) ^§^, ^§§^		0.8473 *
Bronchial asthma	30 (−223 to 254)/−8 (−239 to 263) ^§^, ^§§^		0.7390 *
Skin allergy including atopic dermatitis	61 (−166 to 421)/−9 (−240 to 248) ^§^, ^§§^		0.1638 *
Diabetes mellitus	−60 (−269 to 77)/0 (−225 to 272) ^§^, ^§§^		0.4696 *
Hypertension	−69 (−247 to 183)/0 (−225 to 271) ^§^, ^§§^		0.6409 *
Dyslipidemia	−56 (−220 to 259)/0 (−232 to 268) ^§^_,_^§§^		0.7392 *
Collagen disease	−227 (−368 to 290)/0 (−222 to 261) ^§^, ^§§^		0.3021 *

IQR = interquartile range. *: Mann–Whitney *U* test. ^#^: Spearman’s rank correlation coefficient test. ^§^: yes/no are shown. ^§§^: unknown is excluded. ^§§§^: ever-smoker only.

**Table 3:** Median percentage change in Ab titers from 3 months to 6 months (*N* = 365).

Variable	Percentage Change in Ab Titers, Median (IQR), % ^§^	Correlation Coefficient *ρ*	*p*-Value
Age		−0.028	0.5876 ^#^
Male/female	−25.1 (−36.8 to −12.0)/−31.6 (−42.0 to −20.2)		0.0005 *
Body mass index, median (IQR), kg/m^2^		0.025	0.6274 ^#^
Smoking (ever/never)	−28.4 (−39.7 to −15.5)/−30.3 (−40.7 to −19.0)		0.3051 *
Current smoker/ex-smoker	−31.7 (−40.6 to −18.3)/−27.4 (−40.1 to −16.1) ^§§^		0.3853 *
	ex-smoker vs. never-smoker		0.2914 *
	current smoker vs. never-smoker		0.8809 *
Brinkman Index ^§§^, ^§§§^		−0.034	0.6990 ^#^
Drinking	−29.1(−41.0 to −17.8)/−30.5(−39.1 to −18.1) ^§^, ^§§^		0.777 *
Allergy			
Food	−29.4 (−37.1 to −16.5)/−29.4 (−39.9 to −18.6) ^§^, ^§§^		0.7821 *
Drug	−28.8 (−40.7 to −22.6)/−29.7 (−39.3 to −18.3) ^§^, ^§§^		0.7469 *
Allergic disease			
Allergic rhinitis including pollinosis	−29.2 (−40.9 to −18.1)/−29.6 (−39.6 to −18.0) ^§^, ^§§^		0.8606 *
Bronchial asthma	−31.7 (−40.5 to −19.4)/−29.1 (−40.5 to −17.6) ^§^, ^§§^		0.5521 *
Skin allergy including atopic dermatitis	−30.3 (−44.6 to −25.1)/−29.1 (−39.7 to −17.6) ^§^, ^§§^		0.1677 *
Diabetes mellitus	−27.6 (−32.8 to −11.4)/−29.4 (−40.5 to −18.0) ^§^, ^§§^		0.3617 *
Hypertension	−24.0 (−32.1 to −12.3)/−29.7 (−40.8 to −18.0) ^§^, ^§§^		0.0679 *
Dyslipidemia	−31.7 (−39.2 to −22.8)/−29.3 (−40.5 to −17.7) ^§^, ^§§^		0.7178 *
Collagen disease	−27.0 (−48.6 to −19.1)/−29.6 (−40.3 to −17.9) ^§^, ^§§^		0.889 *

IQR = interquartile range. *: Mann–Whitney *U* test. ^#^: Spearman’s rank correlation coefficient test. ^§^: yes/no are shown. ^§§^: unknown is excluded. ^§§§^: ever-smoker only.

**Table 4:** Age-adjusted median Ab titers and percentage changes in the relationship between sex and smoking.

Variable	Male *n*, Ab, Median (IQR), U/mL Percentage Change, Median (IQR), %	Female *n*, Ab, Median (IQR), U/mL Percentage Change, Median (IQR), %	*p*-Value for Ab Titer Percentage Change
Ever-smokers	70	79	
	−120 (−266 to 152)	−68 (−278 to 193)	0.5709 *
	−25.9% (−38.0 to −12.0)	−30.5% (−42.0 to −18.7)	0.0400 *
Never-smokers	45	171	
	115 (−181 to 519)	46 (−181 to 332)	0.4700 *
	−24.0% (−34.0 to −12.1)	−31.7% (−42.1 to −20.9)	0.0050 *
*p*-value for			
Ab titer	0.0040 *	0.0120 *	
Percentage change	0.7613 *	0.7018 *	

IQR = interquartile range. *: Mann–Whitney *U* test.

## Abbreviations

COVID-19: coronavirus disease 2019; Ab: antibody; IQR: interquartile range; SARS-CoV-2: severe acute respiratory syndrome coronavirus 2.
